# Virulence characteristics of multidrug resistant biofilm forming *Acinetobacter baumannii* isolated from intensive care unit patients

**DOI:** 10.1186/s12879-019-4272-0

**Published:** 2019-07-17

**Authors:** Habib Zeighami, Fatemeh Valadkhani, Reza Shapouri, Elham Samadi, Fakhri Haghi

**Affiliations:** 1 Department of Microbiology, School of Medicine, Zanjan University of Medical Sciences, Zanjan, Iran; 2 Department of Microbiology, Zanjan Branch, Islamic Azad University, Zanjan, Iran

**Keywords:** *Acinetobacter baumannii*, Biofilm, Virulence factor, Integron, REP-PCR

## Abstract

**Background:**

Nosocomial infections and persistence of multidrug resistant biofilm forming *Acinetobacter baumannii* in hospitals has made it as a serious problem in healthcare settings worldwide.

**Methods:**

A total of 100 *A. baumannii* clinical isolates from immunocompromised patients hospitalized in ICU were investigated for biofilm formation, the presence of biofilm related genes (*bap, ompA, csuE, fimH, epsA, bla*_*PER-1*_*, bfmS, ptk, pgaB, csgA, kpsMII*), integron characterization and molecular typing based on REP-PCR.

**Results:**

All isolates were resistant to three or more categories of antibiotics and considered as multidrug resistant (MDR). A total of 32 isolates were resistant to all tested antibiotics and 91% were extensively drug-resistance (XDR). All isolates were able to produce biofilm and 58% of isolates showed strong ability to biofilm formation. All strong biofilm forming *A. baumannii* isolates were XDR. All *A. baumannii* isolates carried at least one biofilm related gene. The most prevalent gene was *csuE* (100%), followed by *pgaB* (98%), *epsA* and *ptk* (95%), *bfmS* (92%) and *ompA* (81%). 98% of isolates carried more than 4 biofilm related genes, simultaneously. Class I integron (67%) was more frequent in comparison with class II (10%) (*P* < 0.05). The REP-PCR patterns were classified as 8 types (A-H) and 21 subtypes. The A1 (23%) and C1 (15%) clusters were the most prevalent among *A. baumannii* isolates (*P* < 0.05). According to the REP-PCR patterns, 23% of all isolates had a clonal relatedness.

**Conclusion:**

Our study revealed the high frequency of biofilm forming XDR *A. baumannii* in ICU patients, with a high prevalence of biofilm related genes of *csuE* and *pgaB.* It seems that the appropriate surveillance and control measures are essential to prevent the emergence and transmission of XDR *A. baumannii* in our country.

## Background

*Acinetobacter baumannii* is an important opportunistic human pathogen that causes a variety of infections as ventilator-associated pneumonia, meningitis, bacteremia, wound and soft-tissue infections, peritonitis and urinary tract infections [1, 2]. High prevalence of multidrug resistant (MDR) *A. baumannii* has emerged as serious problem in healthcare settings in Iran [2, 3]. Persistence and survival ability of MDR *A. baumannii* in various hospital environments and dry condition has made it as a major cause of nosocomial infections worldwide [4]. One of the important factors contributing in chronic and persistence infections and antimicrobial resistance of *A. baumannii* is its capability to colonize and form biofilm on biotic and abiotic surfaces [5]. The biofilm formation rate in *A. baumannii* is 80~91% which is higher than other species (5~24%) [6]. Previous studies have reported a positive relationship between biofilm formation and antibiotic resistance in *A. baumannii* isolates [7]. Several virulence factors involved in bioflm formation of *A. baumannii* such as the outer membrane protein A (OmpA), biofilm associated protein (Bap), chaperon-usher pilus (Csu), extracellular exopolysaccharide (EPS), two-component system (BfmS/BfmR), poly-β-(1,6)-N-acetyl glucosamine (PNAG) and quorum sensing system [4, 5].

Biofilm associated protein (Bap) is a large cell surface protein (854-kDa) and homologous to *Staphylococcal* Bap protein that plays a critical role in cell to cell interactions and biofilm maturation [4, 8]. The 38-kDa outer membrane protein OmpA as major porin of *A. baumannii* plays an important role in attachment and invasion to epithelial cells via interaction with fibronectin. This protein is also involved in serum resistance, biofilm formation and persistence, induction of apoptosis and antimicrobial resistance of *A. baumannii* [1, 4, 8]. Furthermore, previous studies have shown that biofilm formation and attachment of *A. baumannii* to respiratory epithelial cells enhanced in the presence and expression of betalactamase *bla*_*PER-1*_ gene [5]. The CsuA/BABCDE chaperone-usher pilus is necessary for the initiation of biofilm formation on abiotic surfaces. It has been shown that inactivation of the *csuE* gene eliminates pilus production and biofilm formation [4, 9]. The expression of *csu* operon is regulated by a two-component system, *bfm*RS. The *bfm*RS system consists of *bfm*S as a histidine sensor kinase gene which senses environmental conditions and *bfm*R as response regulator encoding gene. According to previous reports, inactivated *bfm*S reduce bioflm formation in *A. baumannii* type strain 17978 [5, 9]. The extracellular polysaccharide poly-β-(1,6)-N-acetyl glucosamine (PNAG) is also involved in biofilm formation, virulence, immune evasion and antibiotic resistance [9].

The present study aimed to investigate the biofilm related genes (*bap, ompA, csuE, fimH, epsA, bla*_*PER-1*_*, bfmS, ptk, pgaB, csgA, kpsMII*), integron characterization and molecular typing based on REP-PCR in multidrug resistant *A. baumannii* isolated from immunocompromised patients hospitalized in ICU.

## Methods

### Bacterial isolates

In our previous study, a total of 100 non-replicate *A. baumannii* clinical isolates were randomly recovered from different specimens including blood, sputum, wound swabs, chest tube secretions and urine from immunocompromised patients with symptomatic clinical infections at least 48 h after ICU admission [2]. Case patients were defined as patients infected by *A. baumannii* according to the Centers for Disease Control and Prevention criteria. The patients who were colonized with *A. baumannii* and immunocompetent patients were excluded. Informed consent and ethical approval was obtained from management of the hospitals prior to the study. The isolates were identified as *A. baumannii* using biochemical tests and PCR targeting the *bla*_*OXA-51*_ gene.

### Antimicrobial susceptibility testing

The antibiotic susceptibility was determined using the disk diffusion method in our previous study [2]. The following antimicrobial disks were used: ampicillin-sulbactam (10/10 *μg*), ceftazidim (30 μg), imipenem (10 μg), gentamicin (10 μg), tobramycin (10 μg), doxycycline (30 μg), ciprofloxacin (5 μg), levofloxacin (5 μg), co-trimoxazole (1.25/23.75 μg), piperacillin (100 μg) and cefepime (30 μg) (MAST, Merseyside, U.K). The results were interpreted according to the CLSI guidelines [10]. Multidrug resistance (MDR) was defined as resistance to at least one agent in three or more categories of antibiotics. *A. baumannii* isolates with resistance to at least one agent in all but two or fewer antimicrobial categories was considered as extensively drug-resistance (XDR) [11].

### Biofilm formation

Biofilm-forming capacity of *A. baumannii* isolates was determined using a microtitre plate assay as described previously [12]. *A. baumannii* isolates were grown overnight in trypticase soy broth (TSB) containing 0.25% glucose at 37 °C. Free cells were removed and biofilms were washed three times with sterile phosphate-buffered saline (PBS) and fixed with 150 mL of 99% (v/v) methanol (Merck). The wells were stained with 1% (w/v) crystal violet for 20 min at room temperature. Crystal violet was dissolved using 33% (v/v) ethanol/acetone (80, 20, v/v) for 20 min and the absorbance was measured at 595 nm. Biofilm formation was scored as follows: **_**, non-biofilm forming (A595 < 1); +, weak (1 < A595 ≤ 2); ++, moderate (2 < A595 ≤ 3); +++, strong (A595 > 3). Reported values are the mean of three measurements.

### DNA extraction

Genomic DNA was extracted from overnight culture of *A. baumannii* isolates using a QIAGEN DNA Mini kit (QIAGEN Inc., Valencia, CA).

### Detection of biofilm related genes

The presence of the biofilm related genes *bap, ompA, csuE, fimH, epsA, bla*_*PER-1*_*, bfmS, ptk, pgaB, csgA* and *kpsMII* was assessed using PCR as described previously [13–18]. The primers sequences are listed in Table 1. PCR was performed using DreamTaq PCR Master Mix (Thermo Fisher Scientific), which contains Taq polymerase, dNTPs, MgCl_2_ and the appropriate buffer. Each PCR tube contained 25 μl reaction mixture composed of 12.5 μl of master mix, 1 μl of each forward and reverse primer solution (in a final concentration of 200 nM), 1 μl of DNA with concentration of 200 ng/μl and nuclease- free water to complete the volume. The PCR was conducted in a Gene Atlas 322 system (ASTEC). PCR was performed according to the following conditions: initial denaturation at 94 °C for 5 min, then, 30 cycles of denaturation (94 °C, 1 min), annealing (the annealing temperature for each gene are listed in Table **1**) for 1 min, extension at 72 °C for 1 min, followed by a final extension at 72 °C for 10 min. The amplified DNA was separated by 1% agarose gel electrophoresis, stained with neutral red (Sigma Aldrich, Germany) and visualized under UV transillumination.

**Table 1:** Primers sequences and the annealing temperatures used in this study

Target Genes	Primers sequences (5–3)	Annealing Temperature(°c)	DNA amplicon Size (bp)	Reference
*epsA*	AGCAAGTGGTTATCCAATCG ACCAGACTCACCCATTACAT	60	451	[12]
*ompA*	CGCTTCTGCTGGTGCTGAAT CGTGCAGTAGCGTTAGGGTA	58	531	[12]
*bla*_*PER-1*_	ATGAATGTCATTATAAAAGC AATTTGGGCTTAGGGCAAGAAA	55	927	[13]
*bap*	TACTTCCAATCCAATGCTAGGGAGGGTACCAATGCAG TTATCCACTTCCAATGATCAGCAACCAAACCGCTAC	55	1225	[14]
*bfmS*	TTGCTCGAACTTCCAATTTATTATAC TTATGCAGGTGCTTTTTTATTGGTC	60	1428	[14]
*ptk*	GGCTGAGCATCCTGCAATGCGT ACTTCTGGAGAAGGGCCTGCAA	57	597	[15]
*pgaB*	AAGAAAATGCCTGTGCCGACCA GCGAGACCTGCAAAGGGCTGAT	57	490	[15]
*fimH*	TGCAGAACGGATAAGCCGTGG GCAGTCACCTGCCCTCCGGTA	60	870	[16]
*csgA*	ACTCTGACTTGACTATTACC GATGCAGTCTGGTCAAC	50	200	[16]
*kpsMII*	GCGCATTTGCTGATACTGTTG CATCCAGACGATAAGCATGAGCA	58	272	[16]
*csuE*	ATGCATGTTCTCTGGACTGATGTTGAC CGACTTGTACCGTGACCGTATCTTGATAAG	60	976	[17]
*intI-1*	CAGTGGACATAAGCCTGTTC CCCGAGGCATAGACTGTA	62	160	[18]
*intI-2*	GTAGCAAACGAGTGACGAAATG CACGGATATGCGACAAAAAGGT	62	788	[18]
*intI-3*	GCCTCCGGCAGCGACTTTCAG ACGGATCTGCCAAACCTGACT	62	979	[18]

### Integron characterization

Class I, II and III integrons were detected using multiplex PCR as described previously [19]. The primers sequences and PCR conditions are shown in Table 1.

### REP-PCR

The REP like elements in the genomic DNA extracted from *A. baumannii* isolates were amplified with the primer pair REP1 5′-IIIGCGCCGICATCAGGC-3′ and REP2 5′-ACGTCTTATCAGGCCTAC-3′ as described previously [20]. Amplification was carried out with an initial denaturation at 94 °C for 10 min, followed by 30 cycles of denaturation (94 °C, 1 min), annealing (40 °C, 1 min), extension (72 °C, 2 min) and a single final extension at 72 °C for 16 min. 20 μl of PCR products were subjected to electrophoresis in 2% agarose gel. Isolates with identical profiles or highly similar profiles (up to 2 bands different) were considered as the same group.

### Statistical analysis

The data were analyzed with SPSS version 17.0 software (SPSS, Inc., Chicago, IL). A chi-square test was used to determine the statistical significance of the data. A *P* value of < 0.05 was considered significant.

## Results

### Characteristics of isolates

Out of 100 *A. baumannii* isolates, 26% were recovered from sputum, 25% from wound swabs, 24% from blood, 15% from urine and 10% from secretions collected from chest tube (thoracic catheter).

### Antimicrobial susceptibility

According to our previous study [2], all *A. baumannii* isolates were resistant to three or more antimicrobial agents and considered as MDR. The highest rate of resistance was detected against ciprofloxacin and imipenem (100%), followed by piperacillin (99%) and cefepime/levofloxacin/ceftazidime (97%). Furthermore, 32% of isolates were resistant to all tested antibiotics and 91% were XDR. The antibiotic resistance patterns of *A. baumannii* isolates are shown in Table 2. The most prevalent pattern was resistance to “ampicillin/sulbactam-ceftazidime-imipenem-gentamicin-tobramycin-doxycycline-ciprofloxacin-levofloxacin-cotrimoxazole-pipercillin-cefepime” with 32% frequency.

**Table 2:** Antibiotic resistance patterns of *A. baumannii* isolates

No. of antimicrobial agents	Antibiotic resistance patterns	Percent (%) of all isolates
5	Ciprofloxacin-Tobramycin-Gentamicin-Imipenem-Ampicillin/Sulbactam	2
7	Ceftazidime-Imipenem-Ciprofloxacin-Levofloxacin-Cotrimoxazole-Piperacillin-Cefepime	2
8	Ceftazidime-Imipenem-Doxycycline-Ciprofloxacin-Levofloxacin-Cotrimoxazole-Piperacillin-Cefepime	5
	Levofloxacin-Ampicillin/Sulbactam-Ceftazidime-Imipenem-Ciprofloxacin-Cefepime-Piperacillin-Cotrimoxazole	8
	Ceftazidime-Imipenem-Doxycycline-Ciprofloxacin-Levofloxacin-Cotrimoxazole-Piperacillin-Cefepime	1
9	Ampicillin/Sulbactam-Ceftazidime-Imipenem-Doxycycline-Ciprofloxacin-Levofloxacin-Cefepime-Piperacillin-Cotrimoxazole	20
	Ampicillin/Sulbactam-Ceftazidim-Imipenem-Gentamycin-Ciprofloxacin-Levofloxacin-Cotromoxazole-Piperacillin-Cefepime	2
	Ceftazidime-Imipenem-Gentamicin-Tobramycin-Ciprofloxacin-Levofloxacin-Cotrimoxazole-Cefepime-Piperacillin	4
10	Ampicillin/Sulbactam-Ceftazidime-Imipenem-Gentamicin-Tobramycin-Ciprofloxacin-Levofloxacin-Cotrimoxazole-Piperacillin-Cefepime	20
	Ampicillin/Sulbactam-Ceftazidime-Imipenem-Gentamicin-Tobramycin-Doxycycline-Ciprofloxacin-Levofloxacin-Piperacillin-Cefepime	2
	Ceftazidime-Imipenem-Gentamicin-Tobramycin-Doxycycline-Ciprofloxacin-Levofloxacin-Cotrimoxazole-Piperacillin-Cefepime	2
11	Ampicillin/Sulbactam-Ceftazidime-Imipenem-Gentamicin-Tobramycin-Doxycycline-Ciprofloxacin-Levofloxacin-Cotrimoxazole-Pipercillin-Cefepime	32

### Biofilm formation assay

All *A. baumannii* isolates were able to produce biofilm; 42 isolates produced moderate biofilm and 58 isolates showed strong ability to biofilm formation.

### Distribution of biofilm related genes

All *A. baumannii* isolates carried at least one biofilm related gene. The frequency of these genes is shown in Table 3. The most prevalent gene was *csuE* (100%), followed by *pgaB* (98%), *epsA* and *ptk* (95%), *bfmS* (92%) and *ompA* (81%). The *csgA* and *fimH* genes were not detected in any *A. baumannii* isolate. Patterns of biofilm related genes in *A. baumannii* isolates are shown in Table 3. Ninety-eight percent of isolates carried more than 4 biofilm related genes, simultaneously. The most common pattern of simultaneous presence of biofilm related genes was “*bfmS-csuE-epsA-bap-kpsMT-ompA-ptk-pgaB*” with 26% frequency (Table 4). Distribution of biofilm related genes among antibiotic resistant *A. baumannii* isolates is shown in Table 5. The most antibiotic resistant isolates carried several biofilm related genes.

**Table 3:** Frequency of biofilm related genes among *A. baumannii* isolates

Biofilm related genes	No = (%) of isolates
*pga*B	98
*ompA*	81
*Bap*	43
*bfmS*	92
*ptk*	95
*epsA*	95
*kpsMII*	57
*bla*_*PER-1*_	2
*fimH*	0
*csgA*	0
*csuE*	100

**Table 4:** Patterns of biofilm related genes among *A. baumannii* isolates

No. of biofilm related genes	Patterns of biofilm related genes	Percent (%) of *A. baumannii*
4 gene	*bfmS-epsA-pgaB-csuE*	2
5 gene	*bfmS- csuE- ompA- ptk- pgaB*	5
	*bfmS- csuE- epsA - ptk- pgaB*	13
	*csuE- epsA - ompA- ptk- pgaB*	3
	*csuE- epsA- bap - ompA- ptk*	2
6 gene	*bfmS- csuE- epsA - ompA- ptk- pgaB*	8
	*csuE- epsA- bap - ompA- ptk-pgaB*	3
	*bfmS- csuE- epsA -kpsMT- ompA- pgaB*	3
	*bfmS- csuE- epsA- bap- ptk- pgaB*	3
7 gene	*csuE- epsA- bap -kpsMT- ompA- ptk-pgaB*	2
	*bfmS- csuE - bap -kpsMT- ompA- ptk- pgaB*	2
	*bfmS- csuE- epsA -kpsMT- ompA- ptk- pgaB*	21
	*bfmS- csuE- epsA- bap - ompA- ptk- pgaB*	3
	*bfmS- csuE- epsA- bap -kpsMT- ptk - pgaB*	2
8 gene	*bfmS- csuE- epsA- bap -kpsMT- ompA- ptk- pgaB*	26
9 gene	*bfmS- csuE- epsA-bla*_*PER-1*_ *-bap -kpsMT- ompA- ptk- pgaB*	2

**Table 5:** Distribution of biofilm related genes among antibiotic resistant *A. baumannii* isolates

Antibiotic resistance	Cefepime (*n* = 97)	Piperacillin (*n* = 99)	Cotrimoxazole (*n* = 95)	Levofloxacin (*n* = 97)	Ciprofloxacin (*n* = 100)	Doxycycline (*n* = 62)	Tobramycin (*n* = 63)	Gentamicin (*n* = 67)	Imipenem (*n* = 100)	Ceftazidime (*n* = 97)	Ampicillin-sulbactam (*n* = 87)
Biofilm related genes											
*csuE*	95	99	95	96	100	62	63	65	100	96	87
*pgaB*	95	98	92	95	98	61	62	63	98	95	86
*ompA*	76	79	74	76	79	62	44	44	79	76	70
*bap*	41	43	41	43	42	31	27	27	43	43	35
*bfmS*	87	90	85	87	90	41	35	33	90	87	77
*ptk*	92	95	90	92	95	60	61	62	95	93	84
*epsA*	92	93	90	92	93	41	35	35	93	92	79
*kpsMT*	55	57	54	55	57	49	30	33	57	55	49
*bla*_*PER-1*_	1	2	1	1	1	1	0	0	2	1	1
*fimH*	0	0	0	0	0	0	0	0	0	0	0
*csgA*	0	0	0	0	0	0	0	0	0	0	0

### Integron characterization

The presence of integrons was confirmed in 67 isolates, of which 67 (67%) and 10 (10%) cases were identified as class I (*intI-1*) and class II (*intI-2*) integrons, respectively. 10% of isolates harbored both *intI-1* and *intI-2*, simultaneously. However, class I integron was more frequent in comparison with class II (*P* < 0.05). Class III integron was not detected in any *A. baumannii* isolate.

### REP-PCR

**Fig. 1:**
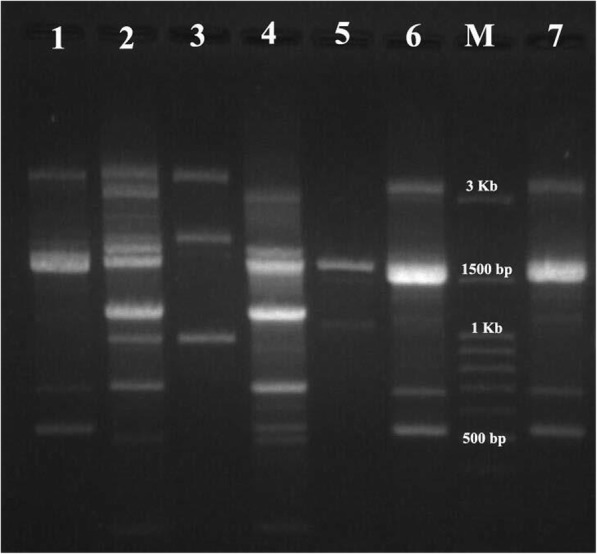
REP- PCR patterns identified among *A. baumannii* isolates examined in this study. Lane 1–7: clinical isolates, Lane M: DNA marker (100 bp)

The patterns generated with REP-PCR contained several bands, ranging in size from 100 to 4750 bp (Fig. 1). These patterns were classified as 8 types (A-H) and 21 subtypes. Among 21 subtypes, 14 distinctive REP-PCR clusters and 7 singleton isolates were inferred from the band patterns. The A1 (23%) and C1 (15%) clusters were the most prevalent among *A. baumannii* isolates (*P* < 0.05) (Table 6). According to the REP-PCR assays, 23% of all isolates had a clonal relatedness. Furthermore, the correlation between REP types (A-H) and biofilm related genes are shown in Table 7. The frequency of biofilm related gene of *bap* was significantly higher among REP type A (P < 0.05). However, no significant association has been found between the REP types and other biofilm related genes.

**Table 6:** REP-PCR patterns among *A. baumannii* isolates

REP Types	Subtypes	Size of REP-PCR products (bp)	Total isolates number (%)
A (3 bands) (24%)	A1	800, 1000, 3000	23
	A2	700, 1500, 2500	1
B (4 bands) (4%)	B1	700, 850, 1000, 1600	3
	B2	900, 1400, 1800, 3000	1
C (5 bands) (27%)	C1	900, 950, 1000, 1500, 2000	15
	C2	800, 1000, 1500, 2500, 3000	9
	C3	700, 1600, 2000, 3000, 4000	1
	C4	550, 700, 800, 1000, 3100	1
	C5	750, 1000, 1900, 2900, 3000	1
D (6 bands) (14%)	D1	800, 1300, 2200, 2500, 3000, 3400	4
	D2	700, 1000, 1900, 2300, 3000, 3300	4
	D3	650, 900, 1900, 2700, 3000, 4750	3
	D4	900, 1000, 1200, 1500, 2000, 3000	3
E (7 bands) (16%)	E1	750,800,1000,1300,1500,2500,3000	6
	E2	650,850,900,1300,2000,2800,3000	9
	E3	250,450,900,1400,2100,2700,3000	1
F (8 bands) (7%)	F1	600,700,900,1200,1600,2000,2800,3200	3
	F2	100,300,400,800,1000,1500,2800,3000	4
G (9 bands) (7%)	G1	300,500,800,900,1600,2300,2800,3200,4300	4
	G2	100,300,500,650,700,900,1100,1700,3000	3
H (10 bands) (1%)	H	100,250,500,700,800,1000,1500,2000,2700,3000	1

**Table 7:** Correlation between REP types and biofilm related genes among *A. baumannii* isolates

REP Types	*pga*B (*n* = 98)	*ompA* (*n* = 81)	*Bap* (*n* = 43)	*bfmS* (*n* = 92)	*Ptk* (*n* = 95)	*epsA* (*n* = 95)	*kpsMII* (*n* = 57)	*bla*_*PER-1*_ (*n* = 2)	*fimH* (*n* = 0)	*csgA* (*n* = 0)	*csuE* (*n* = 100)
A (3 bands) (24%)	24	23	19*	22	24	23	18	2	0	0	24
B (4 bands) (4%)	2	3	1	4	4	4	2	0	0	0	4
C (5 bands) (27%)	27	22	8	25	25	26	17	0	0	0	27
D (6 bands) (14%)	14	10	4	12	14	13	6	0	0	0	14
E (7 bands) (16%)	16	12	6	16	15	15	8	0	0	0	16
F (8 bands) (7%)	7	6	2	5	7	6	3	0	0	0	7
G (9 bands) (7%)	7	5	2	7	6	7	2	0	0	0	7
H (10 bands) (1%)	1	0	1	1	0	1	1	0	0	0	1

*P value of < 0.05 was considered as significant

## Discussion

Persistence and survival of MDR *A. baumannii* in various hospital environments has made it as a major cause of nosocomial infections worldwide [4]. Treatment of infections caused by MDR *A. baumannii* is complicated in Asian countries such as Turkey, India and Iran [2]. In our study, 100% of *A. baumannii* isolates were multidrug resistant. According to previous studies from Iran, the frequency of MDR *A. baumannii* isolates ranged from 32.7 to 93% [21]. Furthermore, 32% of isolates were resistant to all tested antibiotics and 91% were XDR, which agrees with other investigations conducted in Iran [21, 22]. Similar to our results, 98% of *A. baumannii* isolates tested in Saffari et al. study were XDR [22]. It seems that Iran is a hotspot region for the emergence of XDR *A. baumannii* which is serious problem in healthcare settings. Our results also highlight the significance of XDR in *A. baumannii*, because all XDR isolates originated from ICU patients. 100% of isolates were resistant to imipenem and this result is consistent with observations reported from various parts of the world which explain the high risk of failure of carbapenem treatment in *A. baumannii* infections [3, 5, 19, 21, 22]. Recently, there are limited options for treatment of carbapenem-resistant *A. baumannii* infections and colistin and tigecycline are considered the last choice to infection control [2]. Previous studies showed that resistance to ampicillin-sulbactam in *A. baumannii* isolates is increasing in ICU patients [23]. Our study also reports this tendency, with a high frequency of ampicillin-sulbactam resistant isolates (87%), posing another challenge to adequate treatment of *A. baumannii* infections. The high frequency of antibiotic resistance in our survey is the most probably due to the extensive misuse of antimicrobial agents in our country.

The presence of integrons as a primary source of antimicrobial resistance genes within microbial populations is an important factor in the emergence of MDR isolates [22]. In our study, the high frequency of class I integron (67%) in comparision with class II was found (10%), which agrees with other investigations [19, 22]. We also found a significant association between the presence of class I integron and XDR phenotype (*P* < 0.01). Since class I integrons carrying multiple antibiotic resistance gene cassettes, this association is not unexpected. The first report of class I integron in MDR *A. baumannii* from northwest Iran showed that 92.5% of MDR isolates carried class I integron. Significant association between the presence of class I integron and MDR phenotype was reported in their study and other studies [19, 22, 24].

Capability of *A. baumannii* to colonize and form biofilm on biotic and abiotic surfaces is considered as an important factor contributing in chronic and persistence infections [5]. According to our results, all *A. baumannii* isolates were able to produce biofilm and 58% of isolates showed strong ability to biofilm formation. Our results are consistent with previous reports which showed that more than 75% of *A. baumannii* isolates form biofilms [5, 6]. Previous studies have reported a positive relationship between biofilm formation and antibiotic resistance in *A. baumannii* isolates [7, 25]. In our study, all strong biofilm forming *A. baumannii* isolates were XDR. We found significant correlation between strong biofilm formation and XDR phenotype (*P* < 0.01). Several reports have demonstrated that biofilm related genes of *A. baumannii* including *csuE, ompA, bap, epsA, bfmS* were responsible for biofilm development and antibiotic resistance [4–6]. According to our results, the most prevalent gene was *csuE* (100%), followed by *pgaB* (98%), *epsA* and *ptk* (95%), *bfmS* (92%) and *ompA* (81%). Previous studies were also reported the high frequency of *csuE* in *A. baumannii* isolates, so that *csuE* was detected in 100 and 93.8% of isolates in Ghasemi et al. and Youn Sung studies, respectively [4, 6]. The OmpA of *A. baumannii* is probably essential for the attachment to human epithelial cells, development of biofilms and antimicrobial resistance [5]. There was high frequency of *ompA* in our study (81%). Similar results were reported from Thailand and Korea with 84.4 and 68.8% *ompA* positive isolates, respectively [5, 6]. All strong biofilm forming *A. baumannii* isolates were carried *csuE, pgaB, ptk, epsA, bfmS* and *ompA* genes, simultaneously. However, these biofilm related genes were also detected in some moderate biofilm forming *A. baumannii* isolates. According to high frequency of biofilm related genes in *A. baumannii* isolates, the strong biofilm formation was expected. In agreement with previous studies, *bla*_PER-1_ was less common in our study and only 2 isolates harbored this gene [4, 6]. However, recent report from Thailand found *bla*_PER-1_ in 30.2% of *A. baumannii* isolates [7].

The molecular typing of *A. baumannii* is an important tool for determination of genetic and epidemiological relatedness. Repetitive extragenic palindromic sequence based polymerase chain reaction (REP-PCR) is a suitable method for comparison of genetic profiles of *A. baumannii* [26]. According to Grisold et al. [27] and Pasanen et al. [26] studies, the discriminatory power of REP-PCR was found to be sufficiently high and corresponded reasonably well with PFGE. We used REP-PCR technique for *A. baumannii* typing and according to the results, the size of REP-PCR products ranged from 100 to 4750 bp. These patterns were classified as 8 types (A-H) and 21 subtypes. Among 21 subtypes, 14 distinctive REP-PCR clusters and 7 singleton isolates were inferred from the band patterns The A1 (23%) and C1 (15%) clusters were the most prevalent among *A. baumannii* isolates (*P* < 0.05). According to the REP-PCR patterns, 23% of all isolates had a clonal relatedness. Similar to our results, Meshkat et al. was reported that REP-PCR tying of clinical *A. baumannii* isolates generate 10 distinctive clusters (named A to J) and eight singleton isolates. According to their results, up to 94% of all the strains were included in nine distinct clusters and only 6% of them had common roots [28]. In the previous study from Iran, genotypic comparison by REP-PCR revealed that carbapenem resistant isolates belonged to six clones and all clones were spread in the ICUs. Clone A was dominant (30.9%) and clone F had the lowest prevalence (1%) [29]. In our study, the frequency of biofilm related gene of *bap* was significantly higher among REP type A (*P* < 0.05). However, no significant association has been found between the REP types and other biofilm related genes. In study conducted by Dahdouh et al., international clones I and III (IC I, III) was negatively associated with α-hemolysis and strong biofilm formation (*p* < 0.05). But, international clone II and those harboring blaOXA-24-like were positively associated with α-hemolysis, production of strong biofilms, and siderophore production [1].

## Conclusion

Our study revealed the high frequency of biofilm forming XDR *A. baumannii* in ICU patients, with a high prevalence of biofilm related genes of *csuE* and *pgaB.* It seems that the appropriate surveillance and control measures are essential to prevent the emergence and transmission of XDR *A. baumannii* in our country.

## Data Availability

The datasets will not be available on a publically available website, but it may be possible to provide access to anonymized data. Anyone who wants to request the data can contact with Fakhri Haghi, corresponding author.

## Abbreviations

Bap: Biofilm Associated Protein
Csu: Chaperon- usher Pilus
EPS: Extracellular Exopolysaccharide
HLAR: High Level Aminoglycoside Resistance
MDR: Multidrug Resistant
OmpA: Outer Membrane Protein A
VRE: Vancomycin Resistant Enterococci
XDR: Extensively Drug Resistance
